# The impact of cooking and burial on proteins: a characterisation of experimental foodcrusts and ceramics

**DOI:** 10.1098/rsos.240610

**Published:** 2024-09-18

**Authors:** Miranda Evans, Richard Hagan, Oliver J. Boyd, Manon Bondetti, Oliver E. Craig, Matthew J. Collins, Jessica Hendy

**Affiliations:** ^1^ McDonald Institute for Archaeological Research, University of Cambridge, Cambridge CB2 3ER, UK; ^2^ BioArCh, Department of Archaeology, University of York, York, UK; ^3^ Independent; ^4^ The GLOBE Institute, Faculty of Health and Medical Sciences, University of Copenhagen, Copenhagen, Denmark

**Keywords:** palaeoproteomics, ceramics, foodcrusts, experimental archaeology

## Abstract

Foodcrusts have received relatively little attention in the burgeoning field of proteomic analysis of ancient cuisine. We remain ignorant of how cooking and burial impact protein survival, and crucially, the extent to which the extractome reflects the composition of input ingredients. Therefore, through experimental analogues, we explore the extent of protein survival in unburied and buried foodcrusts and ceramics using ‘typical’ Mesolithic ingredients (red deer, Atlantic salmon and sweet chestnut). We then explore a number of physicochemical properties theorised to aid protein preservation. The results reveal that proteins were much more likely to be detected in foodcrusts than ceramics using the methodology employed, that input ingredient strongly influences protein preservation, and that degradation is not universal nor linear between proteins, indicating that multiple protein physicochemical properties are at play. While certain properties such as hydrophobicity apparently aid protein preservation, none single-handedly explain why particular proteins/peptides survive in buried foodcrusts: this complex interplay requires further investigation. The findings demonstrate that proteins indicative of the input ingredient can be identifiable in foodcrust, but that the full proteome is unlikely to preserve. While this shows promise for the survival of proteins in archaeological foodcrust, further research is needed to accurately interpret foodcrust extractomes.

## 1. Introduction

Proteomics has become a valuable tool for identifying food and vessel use in archaeological samples particularly from exceptionally well-preserved remains from frozen [1,2], desiccated [3–5] or waterlogged contexts [6,7], as well as residues in metal vessels [8–10], and calcified residues such as limescale on ceramics [11,12] and dental calculus [13–16]. Due to the tissue and taxonomically specific sequence information proteins can hold, proteomics is particularly useful for the detection of ingredients and cuisine. However, exceptionally well-preserved remains are rare, rendering studies with statistically significant sample sizes and regional comparisons difficult. While protein analysis of human dental calculus can be immensely valuable for understanding consumed diets, it does not necessarily give clear insights into food preparation or links with culinary material culture.

Ceramics associated with food preparation and consumption would be an ideal target sample for proteomics as they are ubiquitous in many contexts. However, the detection of proteins from ceramics themselves has proved challenging, due to either strong binding, or degradation during burial. Proteins have been found to bind strongly to the mineral matrix of ceramic vessels, which likely results in good protein preservation yet renders their extraction challenging [17] without the use of harsh solvents [18]. Conversely, Barker *et al*. [19] concluded that protein content decayed rapidly upon burial, although they did not measure the initial protein content prior to burying their samples, and thus the rapidity of protein loss may be difficult to estimate. Food proteins have been reported from archaeological ceramics [11,20–22] and modern replicas [23,24]. However, there are potential factors aiding the detection of proteins in archaeological cases, such as the inclusion of remnant encrustations [20], or the sampling of ceramic from immediately beneath a limescale deposit [11], which may have provided protection from diagenesis. In the case of Solazzo *et al*. [21], the sherd was from relatively cold conditions in the Arctic coast of Alaska, and contained lipid-rich foods including whale and seal meat, both factors which may have improved protein preservation, although we note that food proteins were not detected in similar ceramics in a later study [25].

### 1.1. Biomolecular analyses of foodcrusts

Given the challenges in extracting proteins from ceramics themselves, foodcrusts may offer a good alternative target sample for proteomic analysis. Foodcrust, sometimes referred to as ‘carbonised residue’ and ‘char’ is broadly defined as ‘amorphous charred or burnt deposits adhering to the surface of containers associated with heating organic matter’ [26]. The prevalence of foodcrusts varies considerably; however, they are particularly abundant in Mesolithic and Neolithic contexts in northern Europe and Eurasia where they are sometimes found on the majority of ceramics within assemblages [27,28]. Examples of archaeological foodcrusts can be viewed in fig. 2 in [29], fig. 1 in [30] and fig. 3 in [31].

**Figure 1.**
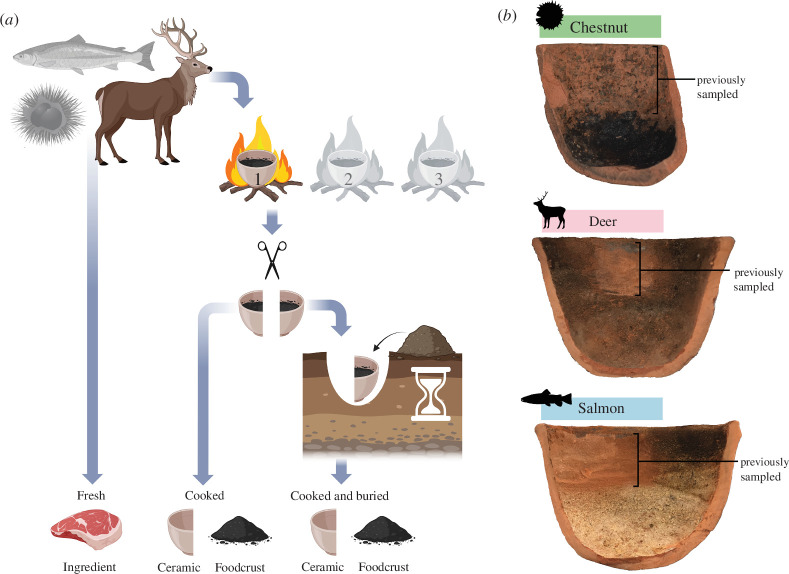
(*a*) Sample creation process (image created with BioRender.com). (*b*) Example of cooked foodcrust samples.

**Figure 2.**
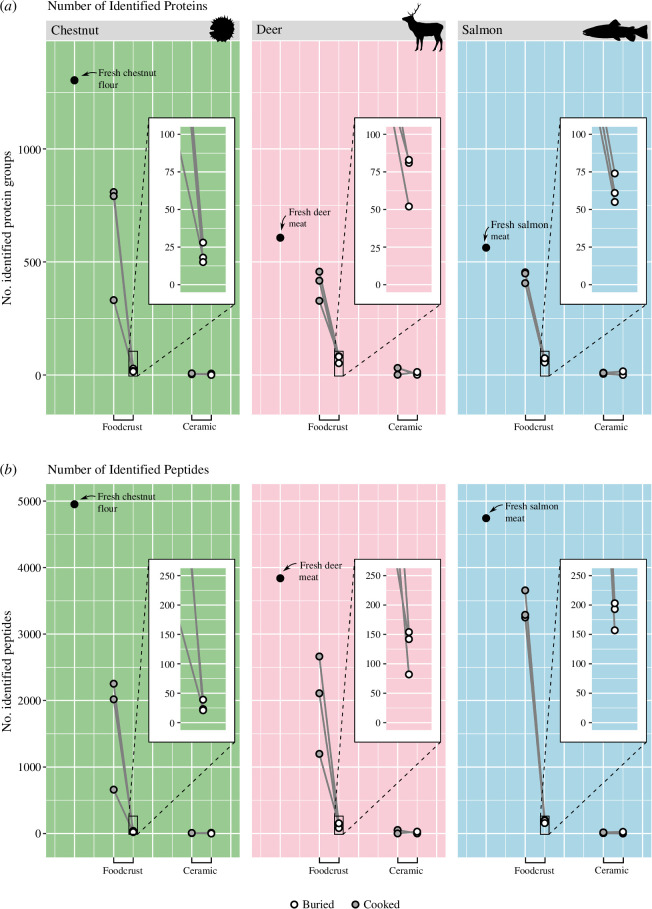
The number of identified proteins (*a*) and peptides (*b*) in foodcrust and ceramic samples cooked with chestnut, deer and salmon. Counts include all peptides/proteins not excluded by filtering steps described above.

**Figure 3.**
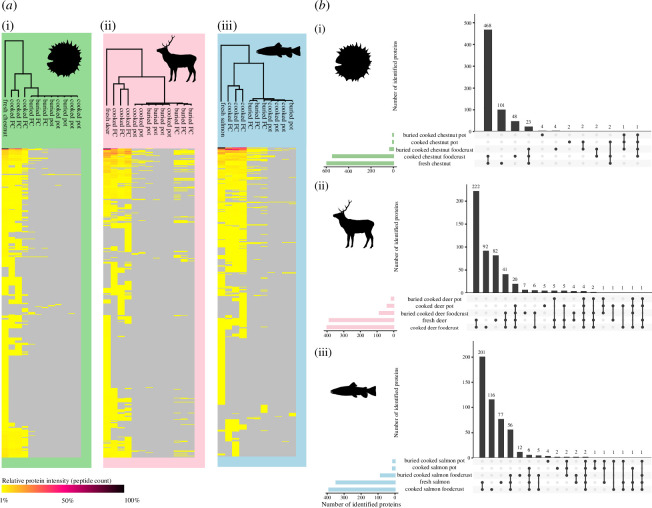
(*a*) Hierarchical cluster analysis of proteins identified in all chestnut (i), deer (ii) and salmon (iii) foodcrust and ceramic samples (Euclidean correlation), created in Perseus v. 1.6.14. (*b*) Upset plot displaying intersection of proteins observed in ceramic, foodcrust and fresh sample categories for chestnut (i), deer (ii) and salmon (iii) samples. Created using UpsetR [65].

Lipid analysis of foodcrusts has considerably improved our understanding of ancient diet and particularly of marine resource utilisation. While frequently applied to ceramics themselves, lipid analysis has also been applied to foodcrusts to detect food and vessel use in assemblages across a vast geography spanning Europe and northern Asia [27,32–38], East Asia [28,39–43] and the Americas [25,44], and to select samples that do not contain aquatic resources for use in carbon dating, which are thus unhindered by the reservoir effect [26]. The formation of foodcrusts is a topic of ongoing research. They are often presumed to be formed by cooking food, although they can also result from the use of fuel for illumination [45] or the production of sealants, moisturisers, adhesives or glues [26,46,47]. A possible correlation exists between foodcrust formation and the processing of aquatic resources, or alternatively these particular lipids may preserve better in foodcrusts than ceramics [27].

Proteomic analysis has recently been applied to archaeological foodcrusts, demonstrating the viability of the technique [29,48], but also has generated questions around protein survival and biases [29,48]. Results so far appear congruent with the association between aquatic resource processing and foodcrusts. Shevchenko *et al*. [29] performed proteomic analysis on four Mesolithic–Neolithic foodcrusts from the site of Friesack 4, Germany. Their results revealed the presence of deamidated fish vitellogenins and parvalbumins in one foodcrust and Suidae collagens detected in another. Lyu *et al*. [48] analysed 21 foodcrusts from the site of Xiawan in southeast China for both lipids and proteins. Their results revealed the presence of potential dietary proteins in five samples, including myosin from large yellow croaker and mandarin fish, and collagen from Caprinae and potentially other mammals [48]. In both of these cases, a low proportion of samples analysed produced dietary results, and the number of dietary proteins and peptides was also low. Despite this initial headway, many questions remain outstanding concerning the survival of proteins in ancient foodcrusts.

### 1.2. Potential preservation biases

The key question is simply the degree to which the proteins identified in ceramics and their residues reflect the original food processed in the vessel. Although proteins indeed become altered through different cooking processes, we remain ignorant of the degree to which those changes impact the detection of proteins in ceramics and foodcrust residues. Similarly, we are also unaware of the impact of burial on the survival of food proteins in these samples.

Compared with proteomics, there is a diversity of published experiments exploring how lipids derived from different ingredients respond to a range of cooking and deposition practises (e.g. [49–55]). For instance, Miller *et al*. [49] demonstrate that absorbed lipids extracted from ceramics represent a long period of use, while surface deposits represent the most recent cooking events. However, as a much younger discipline, such studies are rarer in palaeoproteomics with most experimental studies focused on understanding if proteins survive at all in ceramics, or optimising extraction protocols [18,19,24,56,57] rather than investigating the range of cooking and deposition variables which may impact them (although see [3,12]).

In this study, we characterise the impact of cooking and entrapment in foodcrusts and ceramics, followed by burial on the identification of proteins and peptides from three common Mesolithic foods: *Cervus elaphus* (red deer), *Salmo salar* (Atlantic salmon) and *Castanea sativa* (sweet chestnut), to anticipate results and consider expectations for archaeological interpretations of diet and food preparation practises derived from foodcrusts and ceramics. We aim to identify proteins that persist throughout the cooking process and become embedded in foodcrusts and ceramics, as well as those that persist through burial in soil for six months. Specifically, we examine metrics including peptide and protein count, concentration of different amino acids, peptide length, peptide hydropathicity, peptide isoelectric point, protein thermal stability, protein secondary structure, protein disorder, protein amyloid propensity and relative solvent accessibility (RSA) of the detected peptides in order to identify characteristics of their survival, and we also compare protein with lipid content. Given that proteomics is capable of providing highly specific information concerning ancient ingredients and cuisine, understanding the impact of cooking and burial on the protein content of ceramics and foodcrusts is crucial to accurately interpreting proteomic results of ancient samples. These results provide a maximum baseline for protein recovery from ceramics and foodcrusts under similar conditions.

## 2. Material and methods

### 2.1. Sample creation

Experimental samples included deer meat, salmon meat and chestnut flour, each individually cooked in replica ceramic vessels over the same open fire to induce foodcrust formation, before being split and one half buried. The experiment was repeated in triplicate (figure 1; see also electronic supplementary material, S1 and S2). These were originally created and described previously by Bondetti *et al*. [52] to investigate the formation and diagnostic value of ω‐(*o*‐alkylphenyl)alkanoic acids (APAAs). After lipid extraction, samples were stored at 4°C until protein analysis was performed in summer 2020. Full details of the methodology used for sample creation, protein extraction, and machine analysis can be found in electronic supplementary material S1.

### 2.2. Protein extraction

All samples and machine washes were analysed following an SP3 protein extraction protocol [58,59] adapted for ancient samples [60,61] which can be found on protocols.io (https://doi.org/10.17504/protocols.io.bfgrjjv6), and is routinely applied to archaeological samples [61–63].

### 2.3. Mass spectrometry analysis

The samples were analysed by liquid chromatography tandem mass spectrometry (LC-MS/MS) on an Orbitrap Fusion at the Centre for Excellence in Mass Spectrometry at the University of York. Blank machine washes were run between each sample injection in order to examine and reduce the degree of carry-over between samples.

### 2.4. Data analysis

Samples and machine washes were analysed using Maxquant (v. 2.1.0.0). Peptides were searched allowing for tryptic cleavage, up to two missed cleavages, minimum length of seven amino acids, with both a protein and peptide target false-discovery rate of 1%. Variable modifications included oxidation (M), acetylation (protein N-term), deamidation (NQ), glutamine to pyroglutamic acid and the fixed modification of carbamidomethyl (C) was specified.

All samples were searched against a combined database which included a European red deer proteome (UP000242450), an Atlantic salmon proteome (UP000087266), a Chinese chestnut proteome (UP000737018) and ‘cRAP’, a database of common laboratory contaminants. *Castanea mollissima* (Chinese chestnut) was chosen as a reference database as there was no proteome for *C. sativa* (sweet chestnut) on Uniprot at the time of analysis. To investigate any potential cross-contamination during the outdoor cooking experiment, in the laboratory and due to carry-over in the LC-MS/MS, all samples were searched against all databases, to establish a baseline of cross-contamination. The ‘match between runs’ option was not allowed, given the varying proteomes present as match between runs has been found to falsely inflate peptide identifications [64]. Lowest common ancestor (LCA) was generated for peptides where possible using Unipept Desktop (v. 2.0.0). The data were filtered to remove potential machine carry-over and laboratory contaminants. Full details of this process can be found in electronic supplementary material, S1, and the resulting data can be seen in electronic supplementary material, S3 and S4. It became apparent that cross-contamination occurred during field experiments, which is particularly evident in low protein samples such as ceramic extracts (electronic supplementary material, S5). To minimise the impact of cross-contamination on protein characterisation, only samples with known cross-contaminant peptide concentration ≤2% of the total peptide count were included in the analysis of protein properties (electronic supplementary material S5).

## 3. Results and discussion

### 3.1. Impact of cooking and burial on protein and peptide detection in ceramics and foodcrust

#### 3.1.1. Do proteins preferentially survive cooking and burial in ceramics or foodcrust?

It is immediately apparent that foodcrusts are more likely to harbour preserved proteins than ceramics using the extraction methodology utilised here. Overall, the peptide and protein count for each food was high in the fresh ingredient, reduced slightly in the cooked foodcrust and further reduced (yet still appreciable) in the buried foodcrust samples, with some variation depending on ingredient (figure 2; see also electronic supplementary material, S6) (i.e. <4–8 proteins in chestnut foodcrusts, <24–27 in salmon foodcrusts, <16–33 in deer foodcrusts, supported by >1 peptide spectral match). Statistical testing supports that protein count is similar in fresh ingredients and cooked foodcrust samples (no difference observed at *p* < 0.05, Wilcoxon rank sum test), while a difference in protein count was observed between unburied and buried foodcrusts (Wilcoxon rank sum test, *p* < 0.001; see electronic supplementary material, S7, for all statistical test results). In contrast to the foodcrust, protein and peptide counts were much lower in the ceramic samples, even prior to burial, and remained extremely low after burial (figure 2). This finding was supported by a Wilcoxon rank sum test which did not support a difference in protein count between unburied and buried ceramic samples at *p* < 0.05. This indicates that small but appreciable numbers of food proteins may be detectable in foodcrusts in archaeological samples of similar ingredients and conditions but in contrast, given that few positive protein identifications could be made from buried ceramics, ceramic samples should not be expected to result in positive protein results if a similar protocol is followed.

#### 3.1.2. Why are so few proteins detected in ceramics?

Euclidean clustering revealed that the cooked ceramic samples clustered together with the buried cooked ceramic samples for all food types (figure 3*a*), and statistical testing did not support a difference in peptide count between unburied and buried ceramic samples at *p* < 0.05 (Wilcoxon rank sum test), indicating little or no change. This likely demonstrates that the proteins were too strongly bound to the ceramic matrix to be extracted using the protocol here employed, or alternatively, that even prior to burial, protein identification was already very low in ceramic samples, for instance if protein content had not yet impregnated the ceramic walls or if cooking had rapidly degraded any protein content present in the ceramic. This sharp reduction of both protein and peptide counts in the ceramic samples compared to the fresh ingredients is unsurprising in light of existing published research indicating similar findings [17], and that positive results from ceramics lacking encrustations and under normal preservation conditions have rarely been reported (although see [21–23]). Further work is necessary to devise optimal extraction methods for ceramic-bound protein.

#### 3.1.3. Which proteins survive cooking and burial?

A key aim of this study was to identify the proteins that persist throughout the cooking process and become embedded in foodcrusts and ceramics, as well as those that persist after burial for six months. The most abundant proteins detected in foodcrusts and ceramics (by peptide count) can be seen in table 1. The most abundant proteins (by peptide count) detected in foodcrusts surviving cooking and burial for six months can be seen in table 2. We note that the highest peptide count proteins detected in ceramic samples include several probable contaminants such as keratins, while the highest peptide count proteins in foodcrust tend to contain more genuine ingredient matches. We also note that despite filtering described above, some cross-contamination is observable especially in chestnut samples, likely derived from the field experiments, when samples of different ingredients were cooked over the same open fire.

**Table 1.** The top five most abundant proteins (by peptide count) preserved in foodcrust and ceramic samples after cooking. Data from replicates have been merged. Probable protein source is noted, including where LCA matches to input ingredient, where protein LCA is non-specific and could potentially belong to either input ingredient or another taxonomy, and where protein LCA could not possibly match to the input ingredient and is therefore derived from contamination. This table is based on proteins with the highest peptide count regardless of taxonomic specificity. We note that many muscle proteins are highly conserved, therefore the Unipept LCA for all peptides for which it was available can be viewed in electronic supplementary material, S3. Note: while uncharacterised proteins were the most abundant class, they have been excluded from this table because they represent many different proteins.

		protein	peptide count	unipept LCA	probable protein source
chestnut	**foodcrust**	cupin type-1 domain-containing protein	218	*Castanea crenata* and *Quercus lobata* ^a^	input ingredient
		heat shock protein 70	97	*Prunus avium, Juglans regia* and *Q. lobata* ^a^	non-specific
		starch synthase, chloroplastic/amyloplastic	90	*C. mollissima*	input ingredient
		SHSP domain-containing protein	81	*C. sativa* and *Q. lobata* ^a^	input ingredient
		chitin-binding type-1 domain-containing protein	67	*C. sativa, Q. lobata* and *Salix viminalis* ^a^	input ingredient
	**ceramic**	IF rod domain-containing protein	5	Mammalia	contaminant
		keratin, type II cytoskeletal 5	4	Mammalia	contaminant
		keratin, type II cytoskeletal 75	3	Sarcopterygii	contaminant
		glyceraldehyde-3-phosphate dehydrogenase	2	Craniata	contaminant
		glycogenin-1	2	Artiodactyla	contaminant
deer	**foodcrust**	phosphopyruvate hydratase (2-phospho-ᴅ-glycerate hydrolyase)	238	Mammalia^b^	non-specific
		troponin T, fast skeletal muscle	235	*C. elaphus hippelaphus*	input ingredient
		myosin-1	230	*C. elaphus hippelaphus*	input ingredient
		fructose-bisphosphate aldolase	221	Artiodactyla	non-specific
		alpha-1,4 glucan phosphorylase	196	Mammalia^c^	non-specific
	**ceramic**	globin family profile domain-containing protein	10	Cervidae	input ingredient
		IF rod domain-containing protein	10	Mammalia	non-specific
		keratin, type II cytoskeletal 5	8	Mammalia	non-specific
		myoglobin	5	Mammalia	non-specific
		glyceraldehyde-3-phosphate dehydrogenase	4	root	non-specific
		haemoglobin subunit alpha	4	*C. elaphus hippelaphus*	input ingredient
		phosphopyruvate hydratase (2-phospho-ᴅ-glycerate hydrolyase)	4	Mammalia	non-specific
		superoxide dismutase [Cu-Zn]	4	not available	
salmon	**foodcrust**	glyceraldehyde-3-phosphate dehydrogenase	930	Salmoninae	input ingredient
		phosphopyruvate hydratase (2-phospho-ᴅ-glycerate hydrolyase)	820	Salmonidae	input ingredient
		myosin heavy chain, fast skeletal muscle-like	814	*S. salar*	input ingredient
		creatine kinase	496	*S. salar* and *Oncorhynchus kisutch*	input ingredient
		fructose-bisphosphate aldolase	390	*Salmo* ^d^	input ingredient
	**ceramic**	keratin, type II cytoskeletal 5	7	Mammalia	contaminant
		IF rod domain-containing protein	5	Mammalia	contaminant
		collagen alpha-2(I) chain isoform X3	4	Salmoninae	input ingredient
		collagen alpha-1(I) chain	3	*Salmo*	input ingredient
		keratin, type II cytoskeletal 75	3	Sarcopterygii	contaminant

^a^ Plant Unipept LCA analysis yielded multiple non-target plant taxa, possibly due to database absences.

^b^ One Salmonidae peptide also detected.

^c^ One Neopterygii peptide also detected.

^d^ Three Temnothorax peptides also detected.

**Table 2.** The top five most abundant proteins (by peptide count) preserved in foodcrust and ceramic samples after cooking and burial for six months. Data from replicates have been merged. Probable protein source is noted, including where LCA matches to input ingredient, where protein LCA is non-specific and could potentially belong to either input ingredient or another taxonomy, and where protein LCA could not possibly match to the input ingredient and is therefore derived from contamination. This table is based on proteins with the highest peptide count regardless of taxonomic specificity. We note that many muscle proteins are highly conserved; therefore, in electronic supplementary material, S8, we have noted the most abundant proteins (by peptide count) which match to taxonomic family (Salmonidae, Fagaceae or Cervidae) or lower detected in buried foodcrusts.

		protein	peptide count	unipept LCA	probable protein source
chestnut	**foodcrust**	TATA box binding protein-associated factor (TAF) histone-like fold domain-containing protein	8	root	non-specific
		actin, alpha cardiac muscle 1	6	Eukaryota	non-specific
		cupin type-1 domain-containing protein	6	Fagaceae and *Q. lobata*	input ingredient
		heat shock protein 70	5	root	non-specific
		IF rod domain-containing protein	5	Mammalia	contaminant
	**ceramic**	fast myotomal muscle troponin-T	5	Neopterygii	contaminant
		IF rod domain-containing protein	3	Mammalia	contaminant
		keratin, type II cytoskeletal 5	2	Mammalia	contaminant
deer	**foodcrust**	actin, alpha cardiac muscle 1	29	Eukaryota	non-specific
		globin family profile domain-containing protein	25	Cervidae	input ingredient
		TATA box binding protein associated factor (TAF) histone-like fold domain-containing protein	12	root	non-specific
		elongation factor 1-alpha	11	root	non-specific
		myoglobin	11	Pecora	input ingredient
	**ceramic**	myosin-1	6	Craniata	non-specific
		actin, alpha cardiac muscle 1	5	root	non-specific
		pyruvate kinase	4	Mammalia	non-specific
		creatine kinase	2	Craniata	non-specific
		filamin-C	2	Craniata	non-specific
		ʟ-lactate dehydrogenase	2	Neopterygii	contaminant
		myosin binding protein C1	2	Pecora	input ingredient
		non-selective voltage-gated ion channel VDAC1	2	Mammalia	non-specific
salmon	**foodcrust**	glyceraldehyde-3-phosphate dehydrogenase	71	Salmonidae	input ingredient
		myosin heavy chain, fast skeletal muscle-like	56	Salmoninae	input ingredient
		fast myotomal muscle tropomyosin (Tropomyosin alpha-1 chain)	46	Salmonidae	input ingredient
		phosphopyruvate hydratase (2-phospho-ᴅ-glycerate hydrolyase)	46	Salmonidae	input ingredient
		fructose-bisphosphate aldolase	32	Salmonidae	input ingredient
	**ceramic**	IF rod domain-containing protein	6	Mammalia	contaminant
		collagen alpha-2(I) chain	3	Artiodactyla	contaminant
		keratin, type II cytoskeletal 5	3	Sarcopterygii	non-specific
		keratin, type II cytoskeletal 6A	2	Sarcopterygii	non-specific

#### 3.1.4. Do all proteins have an equal chance of survival?

**Figure 4.**
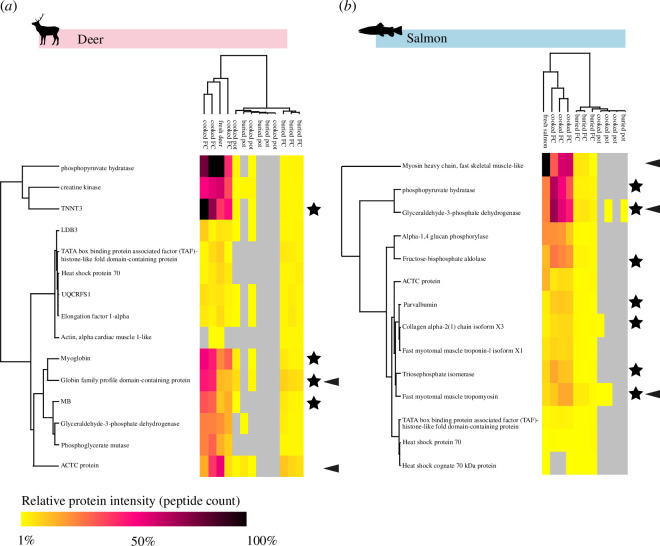
Heat map and dendrogram of proteins present in all buried deer (*a*) and salmon (*b*) foodcrust and ceramic replicates with peptide count >2. Euclidean clustering. Stars indicate proteins which are more peptide-rich in cooked foodcrust samples than in the corresponding fresh ingredient sample. Arrows indicate proteins that are relatively peptide-rich in buried foodcrusts compared to other proteins. Created in Perseus v. 1.6.14.

A central aim of this study was to investigate whether there is a bias towards or against the detection of certain food proteins. We sought to investigate if all proteins followed the same decay trend, i.e. highly numerous in the fresh food, then reducing in number when cooked (and entrapped in foodcrust) and then further reduced when buried. To explore this, hierarchical cluster plots based on highest peptide count grouped by leading razor protein were created (figure 4). For this, only proteins present in all three buried foodcrust replicates with >1 peptide matches were explored, as they were considered to consistently preserve at a quantity appreciable in general palaeoproteomic analysis. The cluster plots revealed that preservation varies by specific protein, and that not all proteins follow the same trend. One notable observation is an increased number of peptide matches to particular proteins in cooked samples when compared with the fresh ingredient, including: Troponin T3, Fast Skeletal Type (TNNT3), myoglobin (MB) and globulin-family profile domain-containing protein (haemoglobin) in deer, and phosphopyruvate hydratase (enolase) and glyceraldehyde-3-phosphate dehydrogenase (GAPDH), and fructose-bisphosphate aldolase in salmon (figure 4, see also figure 3). For example, haemoglobin had a peptide count of 15 in the fresh ingredient, and an average peptide count of 37 in the unburied foodcrust replicates. One explanation for this phenomenon could be the role played by heat in denaturing proteins, partly degrading them and opening them up so that enzymatic cleavage is more efficient. In the buried foodcrusts, while the overall number of proteins is lower than unburied samples, some proteins retained relatively high peptide count compared with others. For example, in the deer samples haemoglobin had an average peptide count of 37 in the unburied foodcrust and 8 in the buried foodcrust, which was relatively high compared with most other proteins. Another example is ACTC protein (actin) which was also relatively abundant (by peptide count) in all three buried foodcrust replicates, and in the salmon samples the comparatively high peptide count proteins included myosin heavy chain fast skeletal muscle-like, GAPDH and fast myotomal muscle tropomyosin. In contrast, while phosphopyruvate hydratase (enolase) is the most abundant protein in terms of peptide count in fresh and cooked deer replicates, it was found in relatively low peptide count in buried foodcrust samples. This reveals that protein preservation is variable: certain proteins persist particularly well in buried foodcrusts while others do not.

#### 3.1.5. Do buried foodcrust results reflect initial ingredient input?

The buried foodcrust samples were intended as analogues of archaeological foodcrusts, and thus to provide a baseline for the extractome that might be expected from archaeological foodcrusts. We investigated the extent to which buried cooked foodcrusts resemble the input protein composition. ‘Upset diagrams’, an alternative to Venn diagrams [66], were used to investigate the proteins shared by each sample type (figure 3*b*). They revealed that for all ingredients, the fresh and cooked foodcrust samples contained the most common shared proteins of any set of samples, indicating that they are compositionally most similar. The largest overlapping group following this was fresh ingredients, cooked foodcrusts and buried foodcrusts in all cases, indicating that despite the reduction of proteins in buried samples, they still somewhat reflect the initial input composition.

To understand whether buried samples revealed the input ingredient taxonomy, Unipept Desktop was used to assign LCA for each peptide where possible (see electronic supplementary material, S3, for peptide Unipept LCAs). Data were then filtered following commonly used proteomic standards (greater than two PSMs to support a protein). In contrast to the buried ceramic samples which harboured no species-specific protein results, all buried deer and salmon foodcrust replicates produced sufficient proteomic evidence to identify the specific input ingredient to a species level in at least one replicate (electronic supplementary material, S6), while two of the chestnut replicates provided tissue-specific evidence with some level of taxonomic specificity (Fagacea or *Quercus lobata*). We note that the plant peptides in particular often yielded matches from *Q. lobata* or other taxonomies related to *Castanea*, which may be due to the absence of many *Castanea* from the Unipept database at the time of analysis. Spectra for the most taxonomically specific peptides in the buried foodcrust samples can be found in electronic supplementary material, S9. Previously, a correlation between the presence of fish products and foodcrusts has been noted [27]. We also note that fish (and deer) proteins are more likely to be preserved in foodcrusts, but the presence of plants in foodcrusts appears underrepresented in protein data. We particularly note that peptides derived from field cross-contamination from salmon and deer were more numerous in buried chestnut foodcrusts than were peptides from the input chestnut proteins (table 2). Therefore, proteomics may not be an appropriate single method through which to address questions of plant processing in antiquity, at least by the methods adopted here. It is apparent that plants generate foodcrusts, but their molecular detection within foodcrusts remains challenging.

These results show that the input ingredient strongly influences the frequency of protein and peptide identifications in foodcrusts and ceramics. Chestnut proteins and peptides were identified less frequently than salmon or deer in buried foodcrusts, despite having higher protein and peptide count in fresh samples, and higher or similar protein and peptide count to deer in cooked foodcrusts (figure 2). This leads us to believe that the comparably low preservation of chestnut in buried samples genuinely reflects their preservation potential relative to the other ingredients rather than other potential explanations such as chestnuts’ lower protein content or the fact that it underwent fewer cooking repeats, indicating an important bias in proteomic analysis of foodcrust residues. While in theory proteomics is capable of detecting proteins from any species represented in reference databases, in practise, it appears that certain ingredients are more likely to be preserved or detected than others. Similarly, this has been observed in the analysis of ancient dental calculus, where a bias towards the detection of milk proteins over other dietary derived proteins has been reported [15]. This has obvious implications on the interpretation of archaeological results, for example rendering plants less visible compared with other ingredients.

Furthermore, in this study, the lack of annotated proteins from some plant species has become starkly apparent. We note that a large number of the peptides identified in the chestnut samples matched to uncharacterised proteins, rendering their analysis difficult. Moreover, the absence of a *C. sativa* proteome in Uniprot at the time of analysis necessitated the use of *C. mollissima* in this investigation—which may impact identifications. This concurs with Hendy *et al*.’s previous comment on the dependence of shotgun proteomics on available databases, and its impact on plant identification [11]. Plants which have much larger proteomes are often absent, particularly for species that are not of current commercial relevance, such as heirloom cultivars. Database absence likely contributes to the lower detection rate of plants in archaeological samples or their detection at higher taxonomic specificity.

### 3.2. Exploration of characteristics enabling protein survival in buried foodcrust samples

#### 3.2.1. Why do particular proteins survive cooking and burial?

Having identified which proteins persist after cooking, foodcrust formation and burial, as well as the overall trend in the number of proteins preserved, we now explore whether these proteins harbour particular characteristics which may facilitate their survival. In this study, it is apparent that degradation is not universal nor linear between different proteins (in contrast to[19]). As discussed above, certain proteins persist particularly well in buried samples while others do not (figure 4), leading us to hypothesise that individual protein properties aid in their preservation. Previously, particular characteristics have been hypothesised to impact protein preservation in or on pottery and other mineral surfaces [17–19,57,67–69]. We wished to explore if there were particular characteristics on either a peptide or protein level that may be impacting the potential preservation of proteins in buried foodcrust samples.

**Figure 5.**
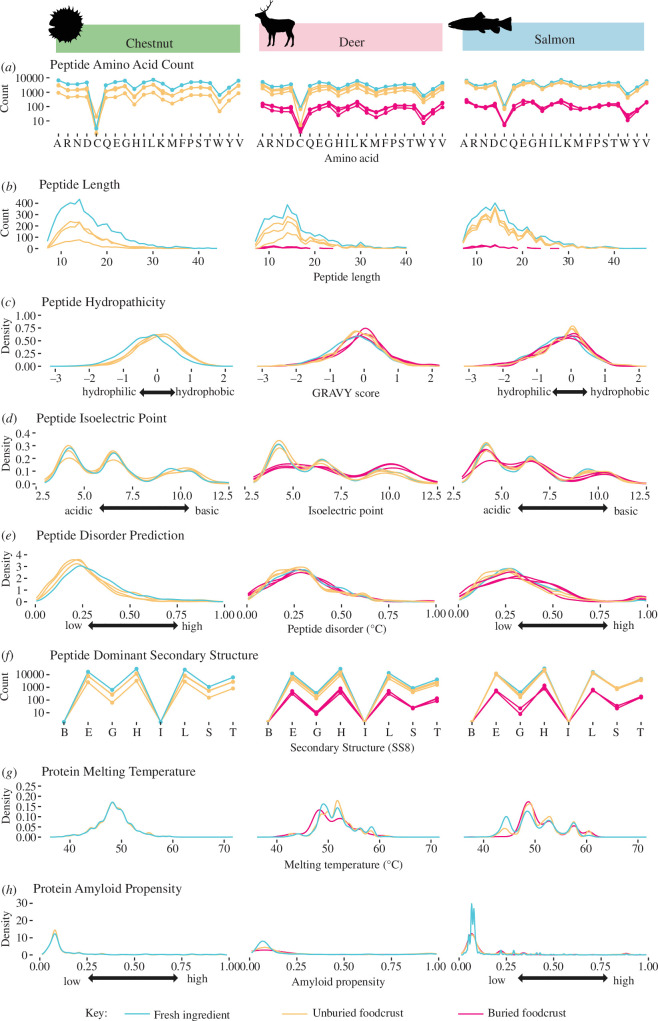
Protein and peptide characteristics for fresh (blue), cooked foodcrusts (orange) and buried foodcrusts (pink) extractomes of chestnut, deer and salmon, including: (*a*) peptide amino acid count (points connected for visibility), (*b*) peptide length, (*c*) peptide hydropathicity, (*d*) peptide isoelectric point, (*e*) peptide disorder prediction, (*f*) peptide dominant secondary structure (points connected for visibility), (*g*) protein melting temperature and (*h*) protein amyloid propensity. Buried chestnut replicates removed due to insufficient sample size and field contamination.

A range of peptide and protein characteristics were investigated. These included concentration of different amino acids, peptide length, peptide hydropathicity, peptide isoelectric point, the sample’s lipid content, protein melting temperature, disorder prediction, amyloid propensity, protein secondary structure and RSA at a given peptide (figure 5). The characteristics of bulk amino acid concentration, peptide length, peptide hydropathicity and bulk deamidation were calculated manually, while various tools were used to calculate the other characteristics. These included: protein thermal stability: DeepSTABp [70]; amyloid propensity: AMYPred-FRL [71]; disorder prediction: IUPred [72]; isoelectric point: IPC [73]; and protein secondary structure and RSA: ‘Predict_Property’, a standalone, offline version of RaptorX Web server [74,75] (https://github.com/realbigws/Predict_Property). A script was written to extract the secondary structure, RSA and disorder prediction for each peptide in the dataset (https://github.com/miranda-e/peptide_property_analyser). All characteristic results were then compiled and displayed using an R script (figure 5). The full details of data analysis are present in electronic supplementary material, S10. While we initially aimed to explore the relationship between protein tertiary structure and preservation, this proved challenging due to the paucity of tools for this analysis, and the poorly annotated or modelled nature of many target proteins. As an alternative way of investigating structural characteristics, we include secondary structure, disorder prediction and amyloid prediction. The characteristics of protein function and cell location were initially investigated through Gene Ontology (GO) terms; however, these explorations were limited by relatively low levels of annotation in the target species’ databases (electronic supplementary material, S11), so this approach was not pursued. Factors other than cooking and burial such as the extraction protocol and data analysis parameters will have also impacted the composition of the extractome; however, all samples have experienced the same extraction and search protocol. Buried chestnut foodcrust data were removed from analysis due to very low peptide counts, and high levels of field cross-contamination (electronic supplementary material, S5), rendering any interpretation with statistical weight challenging.

#### 3.2.2. Proteins that survive in cooked and buried foodcrusts do not have particular amino acid compositions or secondary structures

Previously, the impact of reactive amino acid content has been noted as a factor influencing protein survival [19], as has the impact of higher order structure and the location of a peptide within the structure, which may protect or expose particular peptides [19,68]. Amino acid sequence also has been reported as a driving factor in protein abundance by determining conformational stability and reducing synthesis cost [76]. Bulk amino acid count (figure 5*a*) and peptide secondary structure (figure 5*f*) demonstrated no global change between their fresh, cooked and buried state for any ingredient, indicating that they likely did not play a substantial global role in peptide preservation. Secondary structure is innately linked to a protein’s function and stability, with certain structures being more stable. Secondary structure was collected using the following categories: G: 310 helix; H: alpha-helix; I: pi-helix; E: beta-strand; B: beta-bridge; T: beta-turn; S: high curvature loop; L: irregular (figure 5*f*). Secondary structures have been reported to be distributed across all proteins in the following ratio: alpha-helix, beta-strand, irregular, beta-turn, high curvature loop, 310 helix, beta-bridge, pi-helix = 34:21:20:11:9:4:1:0 [77]. Similar ratios in line with the background distribution were observed in all samples, with only minor variation between input ingredients—meaning that secondary structure does not seem to be a factor in determining which peptides we detect in the extractome. Moreover, as secondary structure and bulk amino acid distribution were no different in cooked or buried samples for any ingredient, it appears that these characteristics do not impact protein preservation.

#### 3.2.3. More hydrophobic peptides are slightly more likely to survive cooking and burial

Previously, the potential role of protein hydropathicity in protein preservation has been hypothesised, whereby hydrophilic proteins leach from ceramics during washing and/or burial [18,19,57]. In this study, we investigated whether peptide hydropathicity (solubility) correlated with peptide count in fresh, cooked or buried samples, with the hypothesis that less water-soluble (hydrophobic) peptides might preferentially survive in buried samples. Grand average of hydropathy (GRAVY) score, a standard measure of protein polarity, was calculated on a peptide level for fresh, cooked foodcrust and buried foodcrust samples (figure 5*c*). Statistical testing supported a difference in GRAVY scores generated across fresh, unburied and buried sample types for the pooled ingredients (Kruskal–Wallis test, *p* < 0.001). Subsequent statistical testing with a Wilcoxon rank sum test supported a difference in GRAVY values between fresh ingredients and their cooked foodcrust counterparts (*p* < 0.001) and between unburied and buried deer foodcrusts (*p* < 0.02). For both salmon and deer, GRAVY score appeared to increase slightly in cooked and buried foodcrusts compared with fresh samples (figure 5*c*), indicating that peptides were generally slightly more hydrophobic in cooked and buried samples than in fresh samples. This result supports previous hypotheses that water leaching over time may reduce soluble protein content in pottery [19,57], leaving slightly more hydrophobic proteins to be detected in higher peptide counts in buried samples, although we note that an increase in hydrophobic peptides was also observed after cooking, indicating that hydrophilic peptides may also be less likely to be entrapped in foodcrust.

#### 3.2.4. Cooking may liberate peptides located deep within the protein’s three-dimensional structure

The potential role of higher order structure and the location of a peptide within the structure, which may protect or expose particular peptides, has been noted [19,68]. RSA is a measure of the exposure of an amino acid within its tertiary structure, and therefore how accessible that residue is to solvents (i.e. amino acids located deeper within the three-dimensional structure are less accessible to solvents). The results revealed that cooked foodcrusts had a higher proportion of peptides with deep RSA (i.e. peptides with amino acids located deep within the tertiary protein structure), than did fresh ingredients, which is most marked in chestnut. One explanation for this is that as tertiary protein structures unfold during denaturation during cooking, peptides which are located deep within the protein structure become more accessible to extraction than they are in uncooked ingredients. This means that in uncooked archaeological samples, we are probably less likely to see deep peptides than in their cooked counterparts. In buried foodcrust samples, the proportion of ‘deep’ RSA peptides continued to decrease in deer samples but increased in salmon samples, providing inconsistent results.

#### 3.2.5. Protein thermal stability may impact peptide survival

Thermal stability is the ability of proteins to resist changes in structure caused by heating. We investigated this characteristic with the hypothesis that thermostable peptides would persist through the cooking, entrapment and burial process. Melting temperature (Tm) is often used as a measure of protein thermal stability. Proteins surviving in buried salmon foodcrusts appeared to be slightly more thermostable than the fresh ingredient and unburied foodcrust, with fewer peptides from proteins of low thermal stability (Tm < 45°C) surviving in buried foodcrust replicates (figure 5*g*). This may indicate that thermally stable peptides were more likely to survive in buried salmon than proteins with lower thermal stability. Conversely in buried deer foodcrust, statistical testing supported a difference in Tm in unburied and buried deer foodcrusts at *p* < 0.05 (Wilcoxon rank sum test) with peptides from less thermally stable proteins appearing to survive better than peptides with higher thermal stability (figure 5*g*), further demonstrating the varied behaviour of different ingredients.

#### 3.2.6. Certain properties may aid in the preservation of particular ingredients or proteins

Some characteristics demonstrated changes after cooking and burial only for particular ingredients. Amyloid propensity and disorder prediction showed changes primarily for salmon, but not in deer samples. Previously, Collins *et al*. [69] speculated that entropic effects would promote survival of flexible molecules that could adapt and bind to the mineral surface. Demarchi *et al*. provided evidence that mineral binding of a small flexible acid rich region was responsible for the persistence of a peptide form of a c-type lectin of African ostrich eggshell into deep time [78]. Most recently, Scott [79] proposed that the robust nature of amyloid fibrils and other factors contributing to protein aggregation may explain the presence of particular proteins and peptides in the archaeological record, noting that dietary proteins persisting in ancient dental calculus are often amyloidogenic. The analysis of intrinsically disordered proteins revealed that in the case of salmon, the proportion of peptides with high disorder prediction slightly increased following cooking and remained high during burial meaning that more flexible peptides become relatively more representative than inflexible ones (figure 5*e*). Deer samples did not display this trend. Statistical testing supported a difference in disorder propensity between all fresh ingredients and unburied foodcrusts at *p* < 0.001 (Wilcoxon rank sum test), while a difference was only observed between unburied and buried foodcrusts in the case of salmon (*p* < 0.05). Similarly, in the analysis of amyloid propensity, while statistical testing supported a difference in amyloid propensity between both deer and salmon unburied and buried foodcrusts at *p* < 0.05 (Wilcoxon rank sum test), visual scrutiny of figure 5*h* only revealed a notable difference for salmon samples. This revealed that peptides which could readily stack were more likely to be detected in buried salmon samples than fresh or cooked (figure 5*h*), potentially indicating that this characteristic plays a role in the preservation of some salmon proteins. In the case of deer, the density of peptides with low isoelectric points (i.e. acidic, water soluble peptides) decreased in the buried samples compared with the fresh ingredient and unburied foodcrust, with a statistical analysis supporting a difference in pI between fresh deer and unburied foodcrust and unburied and buried foodcrust at *p* < 0.001, while the density of peptides with high pIs (basic peptides) increased (figure 5*d*). This demonstrates that acidic peptides survived poorly in buried deer foodcrust, a change not observed in salmon or chestnut. This indicates that certain characteristics may aid in the preservation of particular ingredients or proteins.

While the buried chestnut samples were not included in the broader characterisation analysis due to small sample size, we note that the only two chestnut-specific proteins to survive in buried foodcrust samples (Cupin type-1 domain-containing protein and Chitin-binding type-1 domain-containing protein) are both allergenic. Allergenic proteins are often characterised by their stability, in terms of either their tertiary structure (such as the beta-barrel observed in Cupin-type proteins), or resistance to heat or digestive degradation [80], and have previously been observed to preferentially preserve in ancient dental calculus [81].

The potential role of lipids in creating water-impermeable barriers which may shelter proteins in pottery from forces of degradation has previously been noted [19], although lipids may complicate protein extraction [18]. A vast body of work has explored the impact of organic content on protein and nitrogen preservation in sediments [82–87]. Previously, some of the buried foodcrust replicates were analysed by flame ionisation detector [52], facilitating an opportunity to examine any correlations between protein and lipid content in the same vessel. The total peptide and protein count was compared with these lipid quantities generated by Bondetti *et al*. [52] for each sample. We note that there was a surprising level of variation in lipid concentration between replicates of identical input ingredients and weight. This revealed that in addition to the impact of cooking practise and frequency of use [55], even identically processed foodcrusts are not homogeneous. However, due to the small sample size, it was not possible to reveal the impact of lipid content on protein detection (electronic supplementary material, S12). Future controlled dosing studies would further address the impact of lipid content on protein preservations in ceramics and their residues.

#### 3.2.7. Markers of diagenesis

Peptide length was investigated as a potential indicator of diagenesis from the cooking and entrapment process and/or from burial. Differences in peptide length were observed between fresh ingredients, cooked foodcrusts and buried foodcrusts. The distribution generally indicated that shorter peptides were more likely to be detected in buried foodcrust samples (figure 5*b*). Statistical analysis supports that there is a difference in peptide length between fresh ingredients and buried and unburied foodcrusts (Kruskal–Wallis test, *p* < 0.001), in particular between both fresh chestnut and salmon and their unburied foodcrusts at *p* < 0.05, and between both deer and salmon unburied and buried foodcrusts at *p* < 0.001 (Wilcoxon rank sum tests). Peptide length generally decreased upon cooking, with chestnut providing the most marked reduction. It is notable that long peptides (above ~20 amino acids) were very rarely detected in buried samples of any ingredient. This may demonstrate that long peptides are rarely preserved in buried samples, or alternatively, as long peptides are always less numerous, that the smaller sample size of buried peptides reduces the probability of their detection. We note that this observation, that more degraded samples generally yield shorted peptides, is in line with previous work [88]. As peptide length decreases, it seems likely that the probability of identifying a tissue and taxonomically specific sequence would also decrease. This analysis further revealed differences in the behaviour of the different ingredients.

**Figure 6.**
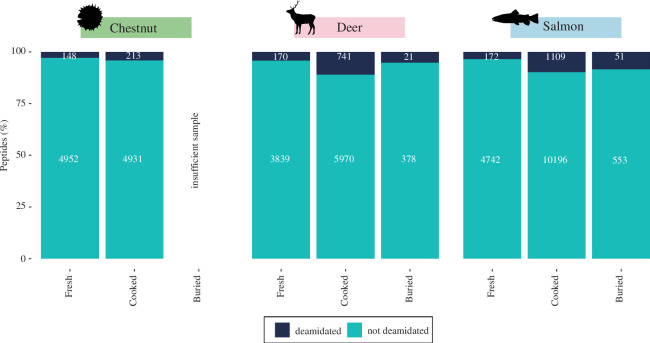
Proportion of deamidated peptides. Note: cooked and buried foodcrust replicates = 3, fresh ingredient replicates = 1.

Deamidation of asparagine and glutamine was explored as a marker of preservational quality [89]. In certain proteins, deamidation has been reported to change protein tertiary structure [90]. In the case of deer and salmon, the highest proportion of peptides bearing deamidation were the unburied foodcrust samples, demonstrating that heating plays a role in deamidation. In the case of salmon and deer, the proportion of deamidated peptides fell after burial (figure 6). Notably, the proportion of deamidated peptides does not continue to increase in the buried state considering that deamidation has been used as an indicator of protein preservation quality, perhaps because the majority of possible deamidations have already occurred during heating, and timescales of chemically mediated deamidation are slow at burial temperatures [91]. This demonstrates that deamidation may not be a good indicator of peptide authenticity where modern contaminants may have undergone heating. Unfortunately, due to the necessary peptide filtration steps discussed in electronic supplementary material, S1, it was not possible to apply deamiDATE here to assess site-specific deamidation. Further investigation of site-specific PTMs may reveal evidence for aspects of food preparation and taphonomy.

#### 3.2.8. Case study: haemoglobin

While no single physicochemical characteristic investigated appears to explain why some proteins survive better than others in buried foodcrusts, it is apparent that these properties may play a role for individual ingredients and proteins. One protein that preserves relatively well in the foodcrust samples is globin-domain containing protein (haemoglobin), so we investigated this protein in greater detail as a case study to understand, on an individual protein level, if particular characteristics may be aiding in its survival. These are likely too complex and varied to be detected broadly, across the whole sample set.

**Figure 7.**
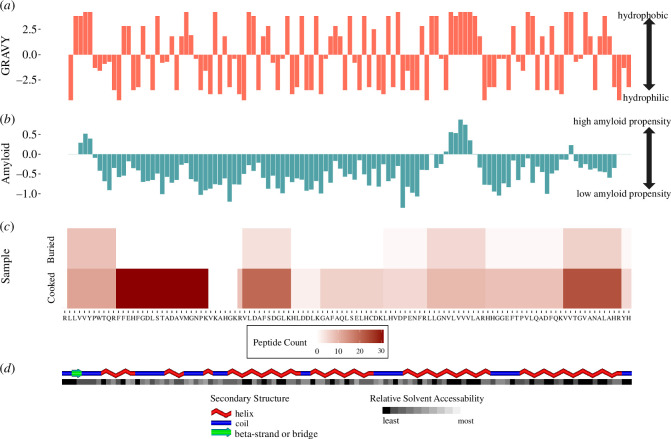
Globin characteristic: hydropathicity (*a*), amyloid prediction (*b*) with heatmaps of peptide counts for each region (*c*) of buried (top) and cooked (bottom) deer foodcrust samples. (*d*) Secondary structure of sequence: blue = coil, red = helix; and RSA: black = completely buried, white = fully exposed (created in POLYVIEW−2D [92]).

GRAVY score, amyloid propensity, secondary structure and RSA were overlaid on top of a peptide count heatmap (figure 7), illustrating that in the case of the globin family protein, the peptides which preserve most commonly in the buried sample are located on the most hydrophobic parts of the protein, and often in regions with high amyloid propensity (figure 7). This demonstrates in the case of globin that hydrophobic (insoluble) peptides appear to be more likely to persist in our buried samples than hydrophilic peptides. However, this means that a complex array of mechanisms, interactions and protein compositions must be impacting the survival of proteins and peptides in buried foodcrusts.

#### 3.2.9. No property single-handedly explains why particular proteins survive cooking and burial

In this study, many protein characteristics were investigated to understand potential explanations for why certain proteins were detected in buried foodcrusts. We observe that the input ingredient was the most influential factor in protein survival, and individual proteins follow different preservation trends. For example, in the foodcrusts created by cooking deer meat, basic peptides seem to preferentially survive the cooking and burial environment. But this trend was not observed for proteins detected from salmon foodcrust. Similarly, proteins with higher thermal stability, disorder prediction and amyloid propensity appear to preferentially preserve in buried salmon foodcrusts, yet not in deer. Some trends were observed across all food types including that slightly hydrophobic peptides are more likely to preserve after cooking and/or burial, that the recovery of longer peptides tended to decrease after cooking and burial, and that deamidation increased following cooking, but not necessarily burial. However, no trend could single-handedly explain why particular peptides and proteins survive cooking and burial. While few trends in protein characteristic were observed across all ingredients, when viewed on an individual protein level, haemoglobin peptides that were slightly hydrophobic and had high amyloid propensity were more likely to preserve in buried foodcrust samples, even though they were not particularly numerous in the unburied sample. This indicates that particular protein or peptide characteristics are indeed involved in determining which proteins/peptides survive in buried samples, but that these complex interactions are likely to vary based on a myriad of factors, and may be obscured when the data are viewed more broadly.

### 3.3. Challenges and future directions

A key goal of this study is to consider expectations for the survival of proteins in foodcrusts and ceramics in archaeological contexts. While the buried foodcrusts in this investigation revealed a number of protein identifications with an informative level of protein and taxa specificity, it is important to note that the samples were buried for only six months and in a temperate climate, and therefore may not be comparable to samples of much older temporal and thermal age. While Barker *et al*. [19] reported that protein content dropped rapidly upon burial and at a slower rate thereafter, the exact rate of dietary protein decay over longer periods of time and in other climatic conditions is poorly understood. The results of this study should therefore be seen as an upper limit to protein preservation, as it may well be that samples of considerable age or from hot climates may not yield confidently preserved proteins, necessitating a cautious approach to the destruction of valuable samples.

In this study, we did not analyse a control sample of the soil in which experimental samples were buried. This was because this experiment was conceived of after the samples used in a different experiment [52] had been excavated, in an effort to maximise the use of and apply comparative approaches to existing material. Steps were taken to mitigate against soil contamination, including removing the most external layer of foodcrust prior to sampling, but we acknowledge that the lack of a soil sample means that it is not possible to filter for any endogenous protein content which may have been present in the soil. However, we note that while endogenous soil proteins may have increased the level of non-specific protein content across all samples, it is unlikely that salmon, deer or chestnut proteins, and even non-taxonomically specific proteins from specific tissues such as muscle proteins, would be present in the soil yet contaminate only certain samples. In future work, the use of a soil sample would enable the testing of background contaminants present in soil.

There are a myriad of factors which could not be investigated in this study, yet may impact protein entrapment and preservation in ancient foodcrusts and ceramics, and thus warrant further investigation. One such factor is the intensity of thermal exposure, for instance with foodcrusts which experienced lower thermal intensity potentially yielding higher protein recovery than those processed at higher thermal intensity. Future work focusing on the impact of increasing thermal exposure to dietary protein detection in ancient foodcrusts may allow more targeted sample strategies. Given the exceptional protein recovery from calcified pottery residues [11,12,93], another factor which will likely impact the preservation of protein in foodcrusts is the level of calcium in the water used during cooking. Here, Yorkshire municipal tap water classified as ‘Hard’ (average calcium content 107.2 mg L^−1^ [94]) was used in the sample creation process, which may have aided in protein entrapment or preservation. Therefore, investigating the impact of a range of calcium concentrations on protein entrapment is a valuable avenue for future research.

In this experiment, the input ingredient itself appeared to play a strong role in the difference in protein preservation/detection between samples. This has implications on ingredient visibility in archaeological interpretations on foodcrust results, particularly for plants. Future investigation of the impact of ingredient mixing on protein preservation will be necessary to understand the full implications of this. Furthermore, further investigation into the impact of protein interactions with other macronutrients such as lipids and carbohydrates on protein preservation is required to fully understand this issue.

We are aware that extraction and LC-MS/MS protocols will have impacted the peptides which are detected. As a result, we are viewing the detected proteins and peptides through a lens of the analytical processes they have been through. Some of these may be taphonomic or cultural such as cooking and burial, but others are inflicted by post-depositional processes, storage, handling and extraction. Untangling the role of each will be imperative in understanding the impact of any one variable. The extraction of proteins from ceramics continues to be a challenge, with ongoing optimisation [17–19,24,56,68,95,96]. The protocol used in this analysis is not optimised for extracting protein from ceramic samples, which may explain their poor recovery here. Ongoing protocol development will hopefully improve the extraction of dietary proteins from ceramics (e.g. [24]). While peptide count and protein count are important metrics used in protein identification in ancient protein studies, in this study, we intended to use Label Free Quantification to quantify differences in protein content between samples; however, unfortunately, this was not possible due to the necessary filtering steps outlined in electronic supplementary material, S1. Future studies would benefit from protein quantification in order to more informatively assess changes in relative and total protein or peptide abundance.

## 4. Conclusion

In this investigation, we sought to explore the utility of proteomic analysis of foodcrusts and ceramics in understanding ancient food preparation, by examining the extent to which the buried foodcrust extractome reflects input ingredients. The results revealed that foodcrusts harboured more preserved proteins than ceramics, and we note, in line with previous studies, that proteins are not easily extracted from ceramic matrices [17,18]. Sufficient taxonomic and tissue-specific identifications were made to detect the relevant ingredient in buried foodcrusts created by cooking deer and salmon meat, and sometimes chestnut flour, demonstrating that ancient foodcrusts may be a viable matrix from which to extract dietary proteins, but that not all ingredients were equal in protein retrieval revealing that input ingredient biases protein recovery. Preservation was not universal between proteins and peptides; those which were most numerous in the fresh meat and flour were not necessarily the most frequently identified in buried samples. These biases have implications on archaeological interpretations of ancient foodcrusts and ceramics, namely that certain ingredients and proteins will be unlikely to be detected in ancient samples. While this study has focused on pottery and foodcrust residues, several of the observations made here regarding protein preservation will likely apply to other areas within the field of palaeoproteomics. It is clear further work is necessary to understand the biases of input ingredients, particularly when they are mixed, and over longer periods of vessel use and burial. Secondly, we attempted to characterise the proteins and peptides retrieved from buried foodcrust samples, to understand physicochemical factors influencing their preservation. While we observed that more hydrophobic peptides were slightly more likely to survive cooking and burial, no property was seen to single-handedly explain why particular proteins/peptides survive in buried foodcrusts, with results indicating that certain properties act on protein preservation in complex ways requiring further investigation, or that characteristics not investigated here may play a role. This study demonstrates the value of experimental analyses to anticipate a maximum baseline of protein results from archaeological samples.

## Data Availability

The mass spectrometry proteomics data, databases used and parameters file have been deposited to the ProteomeXchange Consortium via the PRIDE [97] partner repository with the dataset identifier PXD050001 and 10.6019/PXD050001. Supplementary material is available online [98].
